# MicroRNA-499 Expression Distinctively Correlates to Target Genes *sox6* and *rod1* Profiles to Resolve the Skeletal Muscle Phenotype in Nile Tilapia

**DOI:** 10.1371/journal.pone.0119804

**Published:** 2015-03-20

**Authors:** Pedro G. Nachtigall, Marcos C. Dias, Robson F. Carvalho, Cesar Martins, Danillo Pinhal

**Affiliations:** 1 Department of Genetics, Institute of Biosciences, Sao Paulo State University (UNESP), Botucatu, Sao Paulo, 18618-970, Brazil; 2 Department of Morphology, Institute of Biosciences, Sao Paulo State University (UNESP), Botucatu, Sao Paulo, 18618-970, Brazil; 3 Health Sciences Institute, Federal University of Mato Grosso (UFMT), Sinop, Mato Grosso, 78550-000, Brazil; Ospedale Pediatrico Bambino Gesu', ITALY

## Abstract

A class of small non-coding RNAs, the microRNAs (miRNAs), has been shown to be essential for the regulation of specific cell pathways, including skeletal muscle development, maintenance and homeostasis in vertebrates. However, the relative contribution of miRNAs for determining the red and white muscle cell phenotypes is far from being fully comprehended. To better characterize the role of miRNA in skeletal muscle cell biology, we investigated muscle-specific miRNA (myomiR) signatures in Nile tilapia fish. Quantitative (RT-qPCR) and spatial (FISH) expression analyses revealed a highly differential expression (forty-four-fold) of miR-499 in red skeletal muscle compared to white skeletal muscle, whereas the remaining known myomiRs were equally expressed in both muscle cell types. Detailed examination of the miR-499 targets through bioinformatics led us to the *sox6* and *rod1* genes, which had low expression in red muscle cells according to RT-qPCR, FISH, and protein immunofluorescence profiling experiments. Interestingly, we verified that the high expression of miR-499 perfectly correlates with a low expression of *sox6* and *rod1* target genes, as verified by a distinctive predominance of mRNA destabilization and protein translational decay to these genes, respectively. Through a genome-wide comparative analysis of SOX6 and ROD1 protein domains and through an *in silico* gene regulatory network, we also demonstrate that both proteins are essentially similar in vertebrate genomes, suggesting their gene regulatory network may also be widely conserved. Overall, our data shed light on the potential regulation of targets by miR-499 associated with the slow-twitch muscle fiber type phenotype. Additionally the results provide novel insights into the evolutionary dynamics of miRNA and target genes enrolled in a putative constrained molecular pathway in the skeletal muscle cells of vertebrates.

## Introduction

Skeletal muscle fibers are cylindrical multinucleate cells of the skeletal muscle tissue. In vertebrates, muscle tissue displays two distinct cell-dependent phenotypes, whose categorization is based on the prevalence of fast-twitch or slow-twitch fibers. Fast-twitch fibers are paler-colored muscle cells (white muscle) with larger diameter than slow-twitch fibers. Since fast-twitch fibers have less sarcoplasm and more prominent cross-striping, they are used for forceful and rapid contractions over short periods of time [1,2]. Slow-twitch fibers are small, dark muscle cells (red muscle) that are rich in mitochondria and myoglobin but poor in sarcoplasm, with only faint cross-striping. These cells are designed for slow but repetitive contractions over long periods of time [3]. Dissimilarities between red and white muscle cells in functionality, physiology, and tissue organization are well documented, but the regulation of gene expression programs underlying muscle cell fate, which involves their differentiation and maintenance over time, remain unclear.

In this context, an abundant class of small endogenous non-coding RNA molecules (17–25nt), the microRNAs (miRNAs), has been recognized as posttranscriptional regulators of gene expression. Widely distributed and highly conserved in animal genomes, miRNAs play pivotal regulatory roles and participate in virtually all cellular processes [4]. It is well documented in distinct types of cells from various organisms that miRNA expression varies temporally and spatially, which broadly contributes to producing variable target mRNA and protein expression profiles. Consequently, embryos at distinct developmental stages, as well as tissues and organs from adults, usually show cell-type specific miRNA signatures [5]. In fact, recent studies on mammals have proven that miRNAs are highly enriched or specifically expressed in muscle cells (where they are referred to as myomiRs). MyomiRs act in an intricate gene network regulating cell (fiber) type switching, among other key roles in muscle development and homeostasis [6–8]. Interestingly, experiments carried out on fruit flies (*Drosophila melanogaster)* and zebrafish (*Danio rerio*) have led to similar conclusions, implying that molecular mechanisms regulated by myomiRs may be widely conserved through metazoa [9–12]. However these reports were gathered from early embryos, therefore requiring deep examination of new taxa and at later developmental life stages (differentiated adult tissues) to attest myomiRs conservation.

Another aspect of miRNAs function is that they can modulate the signal transduction pathways by targeting multiple genes (even hundreds of targets *per* miRNA), and a single mRNA displays multiple 3’UTR binding sites for different miRNAs [13]. In this sense, miRNAs-target relationships have evolved in distinct contexts, in which miRNAs may sharpen developmental transitions, act as cell-fate switches, support developmental identity, fine-tune gene expression or insulate target genes expression by reducing transcriptome noise [14,15]. Since myomiRs display a variable set of relationships to target mRNAs, they may be integrated into multiple models of regulation.

Although it is well established that miRNAs promote mRNA cleavage or translational repression [16], the most prevalent effect derived from miRNA activity may differ among organisms. For the majority of target genes investigated so far, the miRNA-mediated effects have not been systematically elucidated. In the present work, we attempt to determine the connection between myomiR expression and muscle cell phenotype in the Nile tilapia, *Oreochromis niloticus*. We also intend to contribute for a better understanding of the miRNA regulatory mechanism (i.e., mRNA degradation or translation repression) potentially operating upon elected target genes

## Results

### Quantitative and spatial expression of myomiRs

**Fig 1:**
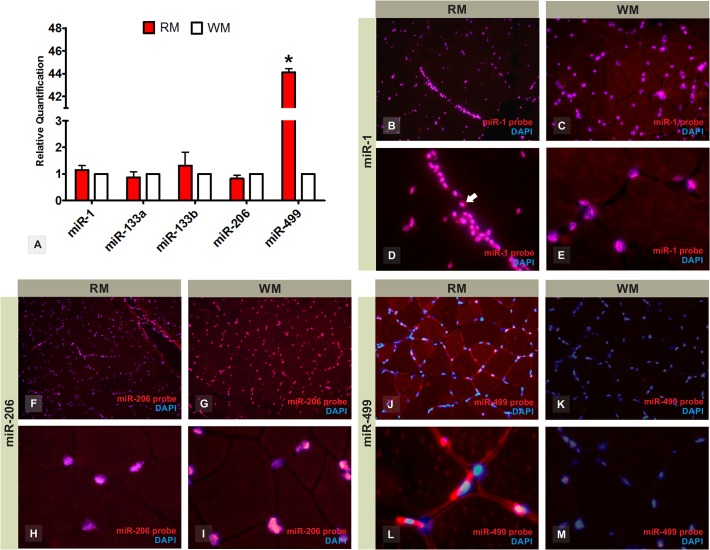
MyomiR expression profile in red and white skeletal muscle fibers of joined males and females adults of Nile tilapia fish. RT-qPCR shows gene expression levels of myomiRs miR-1, -133a, -133b, -206, and -499 between red and white muscle (A). Relative quantification (RQ) values are relative amount of transcripts shown on linear basis. *Statistically significant in comparison to white muscle (P<0.05). MicroRNA fluorescence *in situ* hybridization with LNA probes demonstrates the tissue location of miR-1 at *200x* magnification (B and C) and *400x* magnification (D and E); miR-206 at *200x* magnification (F and G) and *400x* magnification (H and I); and miR-499 at *200x* magnification (J and K) and *400x* magnification (L and M) on red and white skeletal muscle fibers. The miR-1 and miR-206 are clearly expressed in muscle fibers and also connective tissue cells (arrow-head), whereas miR-499 is muscle-fiber-specific. RM = red muscle; WM = white muscle.

Analysis of myomiRs in the red and white skeletal muscle of Nile tilapia by qPCR revealed that miR-1, -133a, -133b, and -206 had a non-significant difference in expression between red and white muscle tissue (p>0.05, Fig. 1A). We also detected non-significant sex-biased differential expression for all myomiRs evaluated (S1 Fig.). Endogenous U6 snRNA and 18S rRNA were correspondingly expressed in red and white muscles proven to be suitable as reference genes for qPCR profiling in skeletal muscle tissue (S1 Table).

Spatial analysis by fluorescent *in situ* hybridization (FISH) pinpointed myomiR expression to the sarcoplasmatic inner edge surrounding the contractile protein core of muscle fibers and at small spots within it. We envision that these are the translation sites of muscle fiber cells where myomiRs would have regulatory action over target mRNAs. Curiously, miR-1 and miR-206 shared an unpredicted tissue expression pattern (Fig. 1B-M). Both are detected not only in nuclear regions of muscle fibers, but also in connective cells of the endomysium and perimysium of Nile tilapia skeletal muscle (Fig. 1D).

Conversely, quantitative analysis revealed that miR-499 had forty-four times higher expression in slow-twitch red fibers than in fast-twitch white fibers (p<0.001, Fig. 1A). The high expression of miR-499 in red muscle was further confirmed by spatial fluorescent analysis (Fig. 1J-M). Such expression profiles were consistent with reports for mammalian, and embryos of zebrafish and medaka (*Oryzias latipes*) [17–19]. Therefore, we directed our attention to investigate miR-499 features in detail, including target genes, potential regulatory mechanisms of target silencing, and functions in adult Nile tilapia skeletal muscle cells.

### miR-499 target prediction and selection

**Fig 2:**
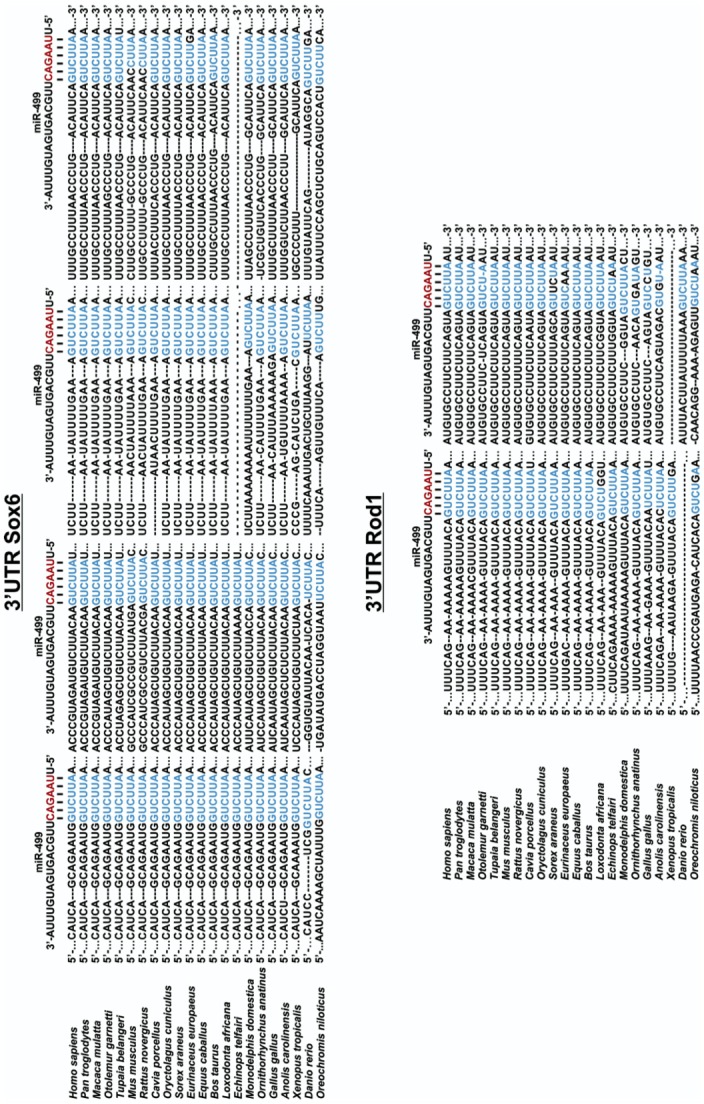
miR-499 binding sites at 3´UTR of *sox6* and *rod1* genes from 22 species of vertebrates. Red letters correspond to the seed regions of miR-499. Blue letters stand for complementary base pairing regions (microRNA recognition elements or MRE) at 3´UTR of *sox6* and *rod1* mRNAs. Dashes are manually inserted gaps, and dots represent continuity of the target mRNAs at both the 5' and 3' ends.

Nearly 200 target genes containing binding sites in their 3’UTR for miR-499 were initially predicted. In our strategy, the 3’UTRs of Nile tilapia were acquired by BLAST searches, and multiple alignments of genome sequences were carried out for Nile tilapia and other vertebrate species. After matching was confirmed, targets were screened using TargetScan. Then, genes with low scores (<1) were removed, leaving a group of twenty-five genes (S2 Table). From the remaining genes, we selected those with higher complementarity to miR-499, conserved 3’UTR binding sites, and known involvement in skeletal muscle biology. After this last filtering step, we selected target genes *sox6* (SRY- *sex-determining region box-6*) and *rod1* (*regulator of differentiation* 1) for better examination, as both genes have known functions in cell proliferation and differentiation [20,21]. We then looked at the nucleotide sequences of the two selected target genes in the genome of twenty-two species of vertebrates. In this survey, *sox6* and *rod1* presented four and two highly conserved miR-499 binding sites in their 3’UTR among vertebrates, respectively (Fig. 2). The minimum free energy (MFE) parameter prediction of all likely miR-499/selected target gene pairings (S2 Fig.) was within broadly accepted ranges and cut-off values [22]. It is worth noting that Nile tilapia is an ectotermic species that lives in temperatures around 25°C, whereas RNAhybrid considers the temperature of endotermic species (37°C) to calculate the MFE. So, interactions predicted by the RNAhybrid would be more strongly supported at lower temperatures, which further endorses the interaction of miR-499 and *sox6* and *rod1* 3’UTRs.

### Target gene transcripts and protein expression in red and white skeletal muscle

**Fig 3:**
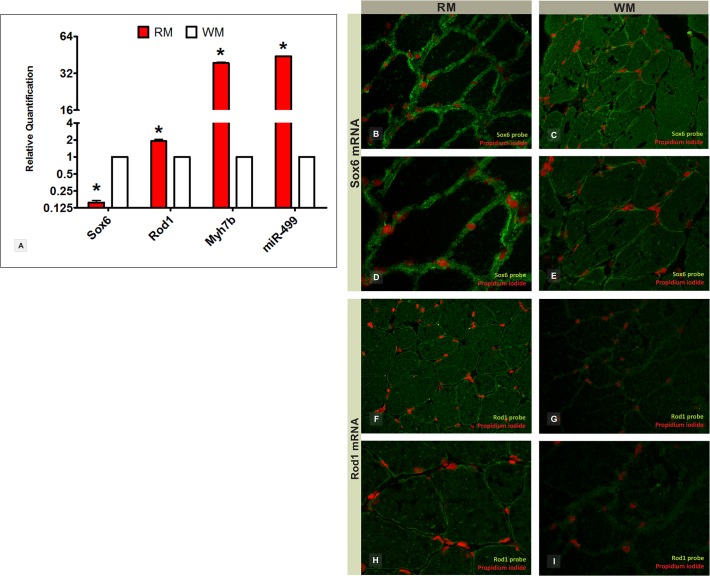
*sox6* and *rod1* gene expression (miR-499 targets) in red and white skeletal muscle fibers of Nile tilapia. RT-qPCR shows gene expression levels of *sox6*, *rod1*, and *myh7b* (miR-499 host gene) between muscle cell types (A). Relative quantification values are shown on Log 2 basis. *Statistical significance in comparison to white muscle (P<0.05). RM = red muscle; WM = white muscle; RQ = relative amount of transcripts. Fluorescent *in situ* hybridization demonstrates the spatial location of *sox6* mRNA at *200x* magnification (B and C) and *400x* magnification (D and E) and *rod1* mRNA at *200x* magnification (F and G) and *400x* magnification (H and I) in red and white muscle fibers.

The quantitative expression analysis of *sox6* and *rod1* mRNA was carried out in red and white skeletal muscles. Because miR-499 is intronic, its host gene *myh7b* (myosin heavy chain 7 beta, or *myh14*), which encodes a slow-twitch heavy chain myosin abundant in the red muscle of fishes and mammals [23–25], was included as positive control. The gene *myh7b* was forty-four times more expressed in the red muscle than in the white muscle of Nile tilapia (Fig. 3A). This result is in accordance with the miR-499 expression, although dissociation between intronic miRNAs and host genes has also been observed [26]. Nevertheless, target genes have shown contrasting mRNA expression levels between cell types. Notably, *sox6* mRNA was seven times less expressed in red muscle compared to white muscle, whereas *rod1* mRNA was two times more expressed in red muscle than in white muscle (*sox6*: RQ = 0.154, p = 0.001; *rod1*: RQ = 2.33, p = 0.001; Fig. 3A). FISH experiments also demonstrated the differential spatial expression of *sox6* and *rod1* mRNA in red and white muscle cells (Fig. 3B-I).

**Fig 4:**
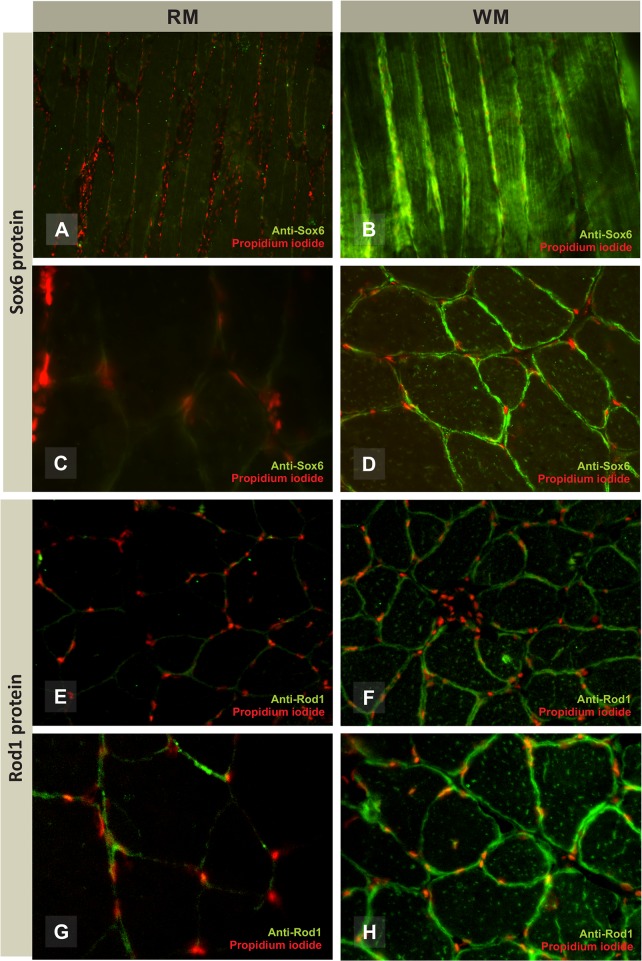
Immunoexpression of SOX6 and ROD1 in red and white skeletal muscle fibers of Nile tilapia. Fluorescent immunostainning demonstrates the SOX6 protein expression at *200x* magnification (A and B) and *400x* magnification (C and D) and ROD1 protein expression at *200x* magnification (E and F) and *400x* magnification (G and H) on red and white muscle fibers. Both of them are expressed only in muscle fiber. RM = red muscle; WM = white muscle.

Regarding protein, both SOX6 and ROD1 were clearly more abundant in white muscle, but they could not be detected in red muscle (Fig. 4). SOX6 and ROD1 were mainly expressed in translation sites of muscle fibers located in the sarcoplasmic inner edge surrounding the contractile protein core of muscle fibers and at small spots within it (S3 Fig.). These proteins were clearly specifically detected in muscle fiber cells with no visible FISH signal in the endomysium (Fig. 4D, H).

From qPCR and immunodetection results, we observed two strongly negative correlations. In white muscle, the low expression of miR-499 correlates with high translation of *sox6* and *rod1* mRNA into proteins. In red muscle, the opposite occurs, as the high expression of miR-499 correlates with low translation, and therefore with a scarce detection of both SOX6 and ROD1 proteins. However, in the latter case, when miR-499 was highly expressed, we found a clear difference in mRNA target levels with a very low expression of *sox6* mRNA. This contrasts with the diminished but still higher *rod1* mRNA levels (Fig. 4). In other words, our findings indicate that miR-499 high expression distinctively connects to the profiles of its target genes, as we detected a prevalence of *sox6* mRNA destabilization contrasting to ROD1 translational decay, which as better discussed below.

### Target genes domains and regulatory network

**Fig 5:**
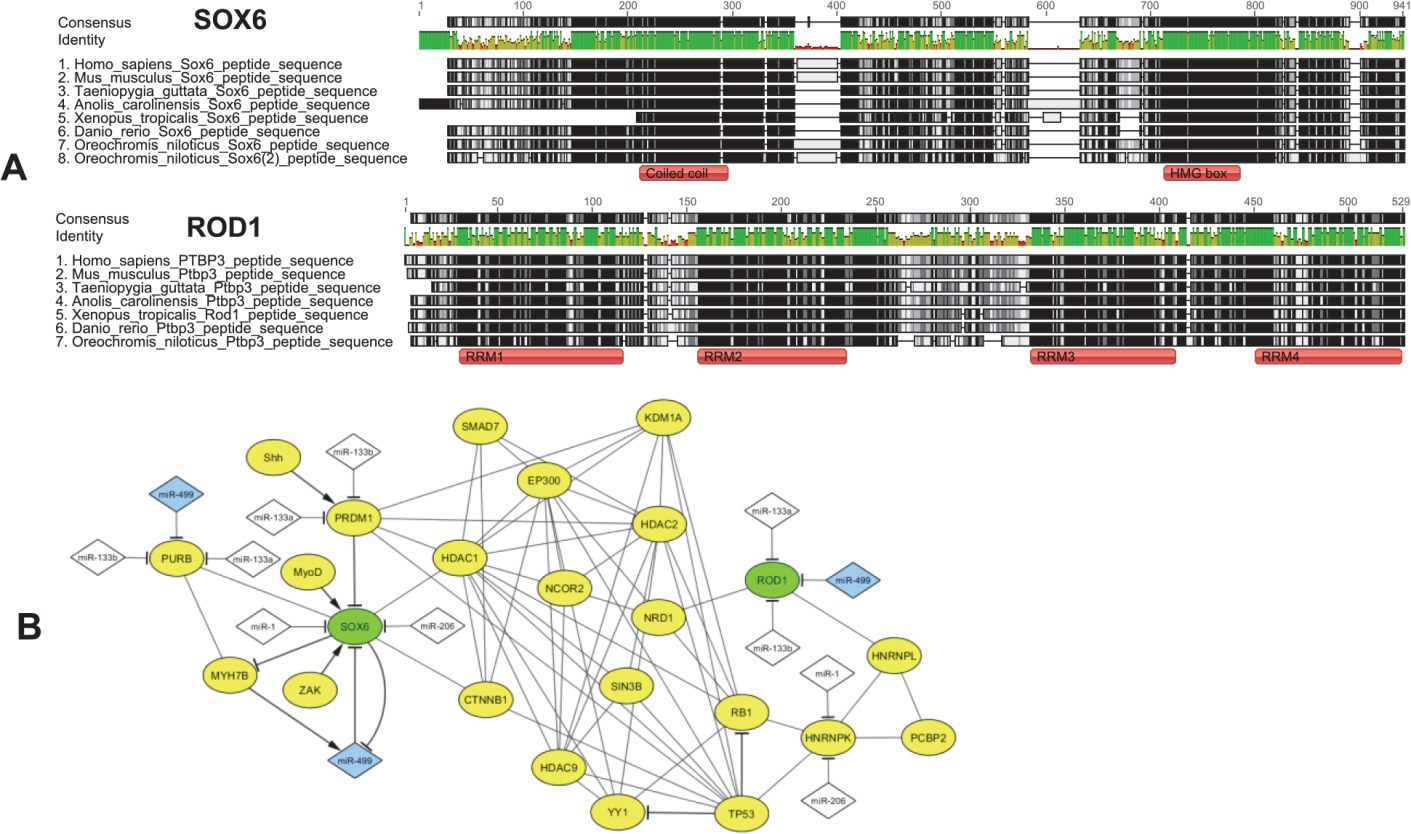
Comparative analysis of protein domains and *in silico* prediction of protein-to-protein interactions (PPI) in the miR-499 gene regulatory network. (A) Alignment of SOX6 and ROD1 sequences retrieved from representatives of the main vertebrate groups. (B) mir-499 gene regulatory network based on PPI filtered using gene ontology. miR-499 are indicated as blue nodes and other myomiRs as white nodes, whereas green nodes correspond to miR-499 target genes evaluated. Transcription factors are shown as dashed-line border nodes, and validated interactions are shown as thick-line bridges.

After measuring the mRNA transcript and protein abundance of SOX6 and ROD1, we looked at their peptide sequences in several vertebrate species. This comparative analysis revealed that both proteins have highly conserved functional domains within the vertebrate genomes examined (Fig. 5A). Once protein functions are determined by the interactions of their functional domains [27], the conservation of SOX6 and ROD1 domains throughout vertebrates would be indicative of conserved protein-protein interactions (PPIs), and consequently, of evolutionary conserved gene regulatory networks. Then, to further explore SOX6 and ROD1 PPIs in muscle cells, we constructed a putative skeletal muscle gene regulatory network based on PPIs and added recognized miR-499 target interactions (Fig. 5B).

We also performed gene ontology (GO) pathway analysis based on GO term enrichment. This analysis supports the relationship of all associated genes in the network as part of the skeletal muscle regulatory network (S3 Table). Fig. 5B shows that the miR-499 network has many interconnections of other myomiRs, proteins, and transcriptional factors associated with distinctive functional categories from regulatory pathways to muscle cell development, proliferation, and differentiation, meaning they might overlap in such critical processes.

## Discussion

Several studies focusing on miRNA gene expression analysis have been carried out on vertebrates with aims to elucidate the real functional significance of myomiRs in distinct fundamental pathways, such as embryonic development, gain or loss of muscle mass, physiology, and maintenance and differentiation of muscle fiber types [28]. Indeed, most myomiR family members such as miR-1, -133, -206, and -499 were reported to be enriched in heart or skeletal muscle, fulfilling distinct roles in transcriptional circuits that control skeletal muscle gene expression [28–30]. Functionally, miR-133 enhances myoblast proliferation during embryonic development, while miR-206 controls the re-innervation and maintenance of neuromuscular junctions and terminal differentiation of myoblasts [31,32]. The miR-1 is critical for myoblast differentiation (as miR-206) and also contributes to the maintenance of the striated phenotype of muscle fibers in adults [29,33]. Whereas, miR-499 expression was shown to be essential for slow muscle fiber type specification and maintenance in some vertebrate species [6,18,19].

In this work, the analysis of myomiR expression in Nile tilapia indicated that miR-1, -133a, -133b, and -206 have equivalently low expression in red and white muscle, and they seem to have no major impact on the adult cell-specific phenotype. Nevertheless, even their basal expression must be important for the control of target *sox6* and *rod1* mRNA and protein levels, as evidenced by the presence, in the human genome, of two miR-133a/b binding sites in *rod1*, as well as one miR-1/206 binding site in *sox6*. Similarly, in zebrafish one binding site each was found for miR-133 and miR-1/206 in rod1, whereas only binding sites for miR-499 where detected in *sox6*. Such findings suggest a putative conjugated fine-tuning effect of myomiRs-mediated repression of *sox6* and *rod1* expression, resulting in the maintenance of the slow contractile pattern of the red skeletal muscle cells. Phenotypic and functional differences between red and white muscle cells could be strongly associated with a remarkably high expression of miR-499 in slow-twitch red fibers. This emphasizes the idea that miR-499 plays key roles in the differentiation and maintenance of the slow-twitch fiber phenotype in Nile tilapia skeletal muscle.

The overexpression of miR-499 in slow contractile machinery muscles was first described in mice by van Rooij and coworkers [6]. A related pattern of miR-499 expression was reported in zebrafish, medaka, and mandarin fish (*Siniperca chuatsi*) [18,19,34]. Furthermore, miR-499 transcription was found to be regulated by *sox6* in a feedback-like response mechanism in zebrafish and mammals [6,26]. In the zebrafish-described mechanism, *sox6* downregulation is mediated by an initial inhibitory action of *prdm1a* that activates the transcription of *myh7b*, together with its intronic miR-499 [18,35]. As a result, miR-499 further decreases *sox6* expression, thus bolstering high miR-499 expression.

In Nile tilapia, we demonstrated for the first time that high miR-499 expression in slow-twitch fibers corresponds with both mRNA target cleavage and translational repression of *sox6* and *rod1* genes. However, remarkably, miR-499 expression occurs at distinctive magnitudes in skeletal red and white muscle cells of Nile tilapia adults. In this sense, our data suggest that miR-499-mediated target repression matches with a reduction in both mRNA and protein levels of *sox6*. In contrast, translational-repression prevails for *rod1*, which suggests decreased translation with a slight mRNA change.

A feasible explanation for ROD1 decay as the major mechanism induced by miR-499 might be related to the limited number of functional miR-499 binding sites on 3’UTR of mRNA (see S2B Fig.). Indeed, a specific miRNA can inhibit gene expression by multiple different mechanisms on individual target genes, and individual miRNAs neither act alone nor target only a unique gene within a network [36]. In this scenario, miR-499 binds on 3´UTR and represses ROD1 translation, probably by competing for cap binding or elF6/60S, inducing mRNA deadenylation or nascent peptide destruction, blocking translation elongation, or inducing a drop-off in ribosome complex [37].

Our findings in fish skeletal muscle are in agreement with those of Hosoda and colleagues [17], who reported that the overexpression of miR-499 in human cardiac stem cells did not affect *rod1* mRNA levels but abrogated ROD1 protein accumulation. Both studies evidenced a same correlational pattern for miR-499 and *rod1* despite of being performed in distinct muscle cells (i.e., cardiac and skeletal muscle). Furthermore, we found a significant reduction in the *sox6* transcripts in slow-twitch fibers of Nile tilapia, indicating that miR-499 might induce *sox6* mRNA destabilization and cleavage, leading to protein decay. One interpretation of these results is that the different types of regulatory mechanisms can be dependent on the number of target sites on 3’UTR of each gene, which is constituted by four target sites on *sox6* and two target sites on *rod1* (Fig. 2).

We detected conserved protein domains and miR-499 binding sites on *sox6* and *rod1* 3’UTR in several vertebrate groups. Such findings give strong evidence for adding *rod1* into the *prdm1a*/miR-499/*sox6* gene regulatory network, described to play important roles for red muscle differentiation and homeostasis in zebrafish [18]. The *rod1* gene would act indirectly on this network via *nrd1*, *ncor2*, *hdac1*, and *rb1* genes. Furthermore, ROD1 is a protein that can bind directly to RNA molecules and modulate the cell differentiation in adult tissues [38]. This protein is also downregulated in slow-twitch muscle, as evidenced in mouse (*Mus musculus*) and human myocardium cells [17]. In the slow-twitch muscle of Nile tilapia. ROD1 was also found to have low expression (Fig. 4E,G). *rod1* transcripts were actually detected in myosatellite cells that are fiber precursor stem cells living in the myocardium and skeletal muscle of vertebrates [17,39]. *rod1* is a gene that negatively regulate differentiation in cellular process through ROD1 binding in RNA target molecules [38]. Thus, considering we found ROD1 was low expressed within the slow muscle and miR-499 was highly expressed, myosatellite cells would differentiate in slow-twitch phenotype muscle fibers,.

We found that the miR-499 regulatory network (Fig. 5) congregates genes associated with skeletal muscle development, differentiation, maintenance, and homeostasis. Our network based on Nile tilapia data expanded upon the previously reported gene regulatory network in zebrafish [18]. Specifically, we could detect many prospective regulatory elements (e.g. *rod1* gene) that could be added into the muscle cell network, which may serve as reference for future studies aimed at a better characterization of the complex scenario involved in the determination of the skeletal muscle cell phenotype. Furthermore, the high conservation of target miRNA recognition element sequences (MREs) into 3'UTR binding sites and the similarity of protein domains verified by *in silico* predictions of PPIs also are noteworthy, considering the hypothesis that the miR-499 network may be functionally widely conserved in higher vertebrates, from fish to mammals. However, future experiments must be carried out to eliminate a possible caveat for the interaction and modulation activities of *rod1* on the *prdm1a*/miR-499/*sox6* gene regulatory network in fish and mammals.

## Concluding Remarks

In summary, we have used quantitative and *in situ* expression associated with gene network and protein sequence analysis to assign a prospective function to miR-499 and target genes *sox6* and *rod1* in regulating fish skeletal muscle cells. Although the knowledge on miR499 function in Nile tilapia can only be assigned by using direct evidence through inhibition-type studies, previous functional studies on other species as well as our spatial and quantitative data (enhanced with protein domain conservation analysis) lead to the establishment of a robust correlation, and it is an indicative of the important role of miR-499 in establishing the white and red muscle tissue phenotype. Also, from protein and mRNA expression data, a distinctive imbalance in mRNA cleavage/translational repression of *sox6* and *rod1* was pioneering reported in fish species. Finally, we recognize that other elements, such as transcription factors, or factors acting on mRNA and protein turnover, may be important in regulating the white and red muscle tissue specific expression of *sox6* and *rod1* [15]. We also can not rule out the existence of a signaling crosstalk among myosatellite, fast and slow muscle cells, given that the intercellular mobility of miRNAs confer robustness to gene expression, which is critical in sharpening the interface between target expression domains [40,41]. For these reasons we proposed a miR-499 gene network (also referring to other myomiRs) that underpins novel associations of genes potentially shaping muscle tissue phenotype, thus providing a likely scenario for the evolutionary dynamics of constrained molecular pathways in the genome of vertebrates.

## Materials and Methods

### Animals and sampling

The Nile Tilapia samples used in this study were obtained from the Royal Fish farm (Jundiaí, Brazil) and maintained at 25±2°C with 12-h light-dark cycles in the Fish Room of the Integrative Genomics Laboratory at Sao Paulo State University—UNESP (Botucatu, Brazil). All animals were handled in accordance with ethical principles for animal research adopted by the local Ethical Commission for Animal Experimentation (CEUA) of the Sao Paulo State University. The experimental protocol of this study was approved by CEUA (protocol number: 352/11).

The adults of Nile Tilapia (16 individuals, 8 males and 8 females) were anesthetized with 50 mg/L tricaine-methanesulfonate (MS-222; Sigma-Aldrich, USA) and sacrificed prior to collection of tissue samples. Red and white muscle samples were processed for real-time qPCR reaction or *in situ* histological detection of microRNAs, target mRNAs, and proteins. Muscle samples were immediately cooled in liquid nitrogen and stored at -80°C until either RNA extraction for qPCR or 10-μm-thick sectioning in a -20°C cryostat for fluorescent *in situ* hybridization (FISH) and immunofluorescence labeling.

### Target prediction and *in silico* analysis of miR-499 gene regulatory network

Prediction of miR-499 targets was performed using the online algorithm TargetScan (http://www.targetscan.org/; release 6.2). Three features were considered for the selection of miR-499 target genes: (a) the number of binding sites on miRNA recognition element sequences (MRE); (b) sequence similarity between miRNA and MRE; and (c) known biological functions of target genes. 3’UTR sequences of putative target genes from 22 vertebrate species were recovered from USCS Genome Browser (https://genome.ucsc.edu/) and aligned in the MUSCLE algorithm using default parameters [42]. The conservation of miR-499 binding sites was then analyzed in MEGA 5 [43]. Beforehand, the 3’UTR of putative target genes from vertebrate species were subjected to BLAST searches against the genome of Nile tilapia for the identification of their orthologs. Also, in order to confirm 3’UTR locations in their respective genes, searches were carried out in Ensembl Genome Browser [44] (Sox6—ENSONIG00000006148; and Rod1—ENSONIG00000012535). The minimum free energy between miR-499 and each target site sequence from 3’UTRs of Nile tilapia was calculated with RNAhybrid [22].

Thus, an alignment of peptide sequences was carried out to evaluate the conservation status of known functional protein domains of SOX6 and ROD1, targets of miR-499, and PRDM1a, putative interaction within SOX6, in vertebrates like fish, amphibian, reptile, bird, and mammal. The subset of alignment was first performed in the MUSCLE algorithm and then verified and annotated in Geneious (Biomatters, Ltd). Due to the high conservation level found in protein peptide sequences, we constructed a gene network from fish data according to our brief findings of high conservation levels between muscle regulatory proteins in vertebrates and also based on human computational data available on String 9.05 database search (http://string-db.org/). Human information was preferably recovered once interactions and proteins are better elucidated and annotated in the human genome.

In this way, the gene interaction network was built to find known and predicted protein-protein interactions (PPI) on regulatory genes linked to biological pathways in the skeletal muscle tissue. Sequences of prdm1a, *sox6*, and *myh7b* genes were uploaded to a String according to their known contribution for the prdm1a/miR-499/*sox6* gene regulatory network, as previously described [18]. After the insertion of *rod1* and other gene partners into the biological network, the computational data source was exported and manually edited in Cytoscape 3.0.2 (http://cytoscape.org). In order to detect only muscle-related proteins in the predicted network, we analyzed the putative enriched terms in the “Biological Process” category in Gene Ontology by using the BiNGO app implemented on Cytoscape. No muscle-related proteins were trimmed out from the network.

### RT-qPCR analysis of myomiRs and target genes

RT-qPCR was applied to evaluate the expression levels of miR-1, -133a, -133b, -206, and -499, along with target genes *sox6* and *rod1*, in red and white muscle samples of male and female specimens of Nile tilapia adults. These myomiR family members where choosen based on their distinct roles in transcriptional circuits that control skeletal muscle gene expression in other vertebrate species. The total RNA was extracted from snap-frozen specimens using Tri Reagent (Invitrogen/Life Technologies, CA, USA). Muscle samples were mechanically dissociated in 1 mL of ice-cold Tri Reagent using a T25 Digital UltraTurrax homogenizer (IKA, China). Total RNA samples were solubilized in RNase-free water (Sigma Aldrich, USA) and quantified by measuring UV-absorbance at 260 nm using a NanoDrop 1000 (Thermo Scientific, USA). RNA purity was ensured by obtaining A_260/280_ ratio >1.8. The total RNA samples were then treated with TURBO DNase (Ambion, USA) to ensure no genomic DNA contamination. RNA quality was assessed using an Agilent 2100 Bioanalyzer system (Agilent Technologies, USA), and only samples with RIN (RNA integrity number) >8.0 were used for RT-qPCR experiments.

The expression levels of myomiRs and target mRNA genes were measured in triplicate on a StepOnePlus Real-Time PCR System (Life-Technologies, USA) following MIQE guidelines [45]. MyomiRs were detected by miRNA cDNA synthesis (reverse transcription) and real-time qPCR amplification using TaqMan MicroRNA Assays (Life-Technologies, USA) following the manufacturer´s protocol. These standard qPCR assays contain probes and primers designed for zebrafish miRNAs (miR-1, -133a, -133b, -206 and -499), which are known to be mature miRNAs that are highly conserved among vertebrates.

The mRNA expression levels of miR-499 target genes *sox6* and *rod1* as well as gene *myh7b* (miR-499 host gene in vertebrates) were detected by total cDNA synthesis (reverse transcription) using a High-Capacity cDNA Reverse Transcription kit (Life Technologies, EUA), followed by real-time qPCR amplification using TaqMan Gene Expression Custom Assays (Life-Technologies, USA) according to the manufacturer´s protocol. These qPCR assays were customized to contain probes and primers designed on exons sites of the investigated genes (highly conserved in fish), which were manually annotated on the Nile tilapia genome (available at Bouillabase.org) with Geneious software. The accession numbers of reference sequences used to recover the genomic sequences of targets on NCBI were XM_001335708 (*myh7b*), XM_003440147 (*rod1*), and XM_003442295 (*sox6*). The two genes, the U6 snRNA (small nucleolar RNA), and the 18S rRNA (ribosomal RNA) were used for miRNA and target gene normalization, respectively, in all qPCR reactions. The sequence of all primers and probes used in this work are listed in S4 Table.

### Efficiency and quantitative expression data analysis

Analysis of qPCR experiments was carried out with LinRegPCR and REST softwares [45,46]. LinRegPCR was used to assess the efficiency of each individual qPCR reaction and represented the gold standard method for quantitative expression experiments using a probe-based approach. This efficiency calculation method is considered a good estimator for the “real efficiency,” because data evaluation is calculated in the exponential phase of PCR amplification [46]. All reactions with efficiency > 0.750 were approved for subsequent analysis (S5 Table). REST was used to calculate the individual expression levels by “Pair-Wise Fixed Reallocation Randomization Test” and relative quantification (RQ). Statistical significance was accepted for p<0.05.

### Fluorescent *in situ* hybridization detection of miRNAs and mRNAs

The miR-1, miR-206, and miR-499 were detected by fluorescent *in situ* hybridization (FISH) with miRCURY LNA microRNA Detection probes (Exiqon, Denmark). Standard *in situ* hybridization reactions were performed as described [47]. We did not analyze the muscle-specific microRNAs miR-133a and miR-133b by *in situ* hybridization due to their genomic organization into clusters miR-1/133a and miR-206/133b [48]. FISH reactions were performed on 10-μm-thick cryosection slides using LNA-specific probes and signal amplification detected with an HNPP Fluorescent Detection Set (Roche, Germany) following manufacturer´s protocol. Nuclear counterstaining was assessed by DAPI. Controls were performed using LNA miR-scrambled and LNA U6 snRNA probes (Exiqon, Denmark) (S4 Fig.).

We also performed fluorescent *in situ* localization of *myh7b*, *sox6*, and *rod1* genes on 10-μm-thick cryosection slides from the red and white muscle of Nile tilapias. Specific DNA biotin-labeled probes complementary to the target gene sequences were synthetized by PCR with biotin-labeled nucleotides using tilapia red and white muscle pool RNA template. Like the primers and probes designed previously, the primers used to generate DNA probes for this step were constructed in exon intersection sites (CDS sequence) of the target genes manually annotated on the Nile tilapia genome. We used the forward 5′-TGGCAGGTGTATCCCCGAGAGC-3′ and reverse 5′-AGCTCCTGTGGTGGCTGCCT-3′ primers for tilapia *myh7b* probe synthesis; forward 5′-GCTGCACAGTCTGCTTCATG-3′ and reverse 5′-TGTGGGGAAGTGGAATTGTTC-3′ for tilapia *sox6*; and forward 5′-TGCATCCCATCGCGGGTCCT-3′ and reverse 5′-TGGTGGGTGTGGCTGAGGTGT-3′ for tilapia *rod1*. Thus, probe synthesis was carried out by adding 0.00672 mM of 16-biotin-dUTP (Roche, Germany) into a PCR master mix containing 3.2 mM dATP, 3.2 mM dCTP, 3.2 mM dGTP, 1.92 mM dTTP, 0.252 mM of each forward and reverse specific primer, 1x PCR buffer, 2.5 mM MgCl_2_, 1 unity of Taq Platinum, and template-mixed cDNA from red and white muscle samples of Nile tilapia. The PCR products (DNA probes) were purified by ExoStar (Illustra, USA) and sequenced by Sanger to confirm the nucleotide sequence composition [49]. The fluorescent *in situ* detection of target genes was performed on 10-μm-thick cryosection slides using the DNA-specific probes and signal amplification identified by an HNPP Fluorescent Detection Set (Roche, Germany) following the manufacturer´s protocol. Nuclear counterstaining was done with propidium iodide (Vector Laboratories Inc., USA) at room temperature for 5 minutes. Images were acquired with an Olympus BX61 fluorescence microscope coupled to a DP71 digital camera (Olympus, Japan).

### Immunofluorescence detection of SOX6 and ROD1 proteins in muscle cells

We used the immunofluorescence (IF) detection of SOX6 and ROD1 to confirm the downregulation of miR-499 target proteins in slow-twitch red muscle fibers of Nile tilapia. Negative controls lacking primary and/or secondary antibodies were also investigated to ensure maximum specificity. IF was performed on 10-μm-thick cryosection StarFrost *plus* slides using primary antibodies polyclonal rabbit-IgG anti-SOX6 (1:100, Sigma-Aldrich, USA) and polyclonal rabbit-IgG anti-ROD1 (1:75, Abcam, USA). The permeabilization of tissue membranes was performed by digestion with proteinase K (10mg.ml^-1^) at 37°C for 5 minutes, and non-specific antibody attachment was blocked with nonfat milk diluted to 1% in PBS at room temperature for 60 minutes. The slides were then incubated overnight at 4°C with the primary antibodies as previously described.

After saline buffer washes, the slides were incubated with secondary antibody biotin-conjugated anti-rabbit-IgG (1:200, Sigma-Aldrich, USA) at room temperature for 60 minutes. Detection of antibody binding sites was done by repeated streptavidin FITC-conjugated (1:100, Life-Technologies, USA) and anti-FITC biotin-conjugated (Sigma-Aldrich, USA) addiction in slides at 37°C for 30 minutes. Nuclear counterstaining was done with propidium iodide (Vector Laboratories Inc., USA) at room temperature for 5 minutes. Images were acquired with an Olympus BX61 fluorescence microscope coupled to a DP71 digital camera (Olympus, Japan).

## Supporting Information

S1 Fig Sex-biased expression comparison of myomiRs in red and white muscle cells of Nile tilapia. RT-qPCR shows gene expression levels of myomiRs miR-1, -133a, -133b, -206, and -499 between males and females in red muscle (A) and white muscle (B). RM = red muscle; WM = white muscle.
(TIF)

S2 Fig Bond strength between miR-499 and target sites at 3´UTR sequence of *sox6* and *rod1* genes from Nile tilapia. Minimum free energy (MFE) in which green lines represents the miR-499 sequence and red lines represent 4 microRNA recognition elements (MRE) on *sox6* mRNA (A) or 2 MREs on *rod1* mRNA (B).
(TIF)

S3 Fig ROD1 expression in the transitional interface of red and white muscle cells. Fluorescent immunostainning demonstrates the difference of ROD1 expression in red, intermediate, and white muscles, *200x* magnification. RM = red muscle; IM = intermediate muscle; WM = white muscle.
(TIF)

S4 Fig Experimental controls of fluorescence *in situ* hybridizations. U6 snRNA and scramble LNA probes detection at red (RM) and white (WM) muscles.
(TIF)

S1 Table Expression of endogenous gene controls by qPCR. U6 snRNA and 18S rRNA were equivalently expressed in red and white skeletal muscle of Nile tilapia.
(TIF)

S2 Table Predicted target genes for miR-499. List of 20 muscle-related predicted target genes for miR-499 in the human genome.
(TIF)

S3 Table Top 20 enriched GO terms associated with skeletal muscle regulatory pathways. Gene Ontology terms and corresponding genes related to predicted miR-499 gene regulatory network in vertebrates.
(TIF)

S4 Table Primers used for the analysis of miR-499 target genes expression. (TIF)

S5 Table Values of efficiency of qPCR reactions as calculated in LinRegPCR. (TIF)
