# Supplementary figures and images for: MicroRNA-499 Expression Distinctively Correlates to Target Genes sox6 and rod1 Profiles to Resolve the Skeletal Muscle Phenotype in Nile Tilapia
Source: PLoS One. 2015 Mar 20;10(3):e0119804. doi: 10.1371/journal.pone.0119804 (PMC4368118; doi:10.1371/journal.pone.0119804)

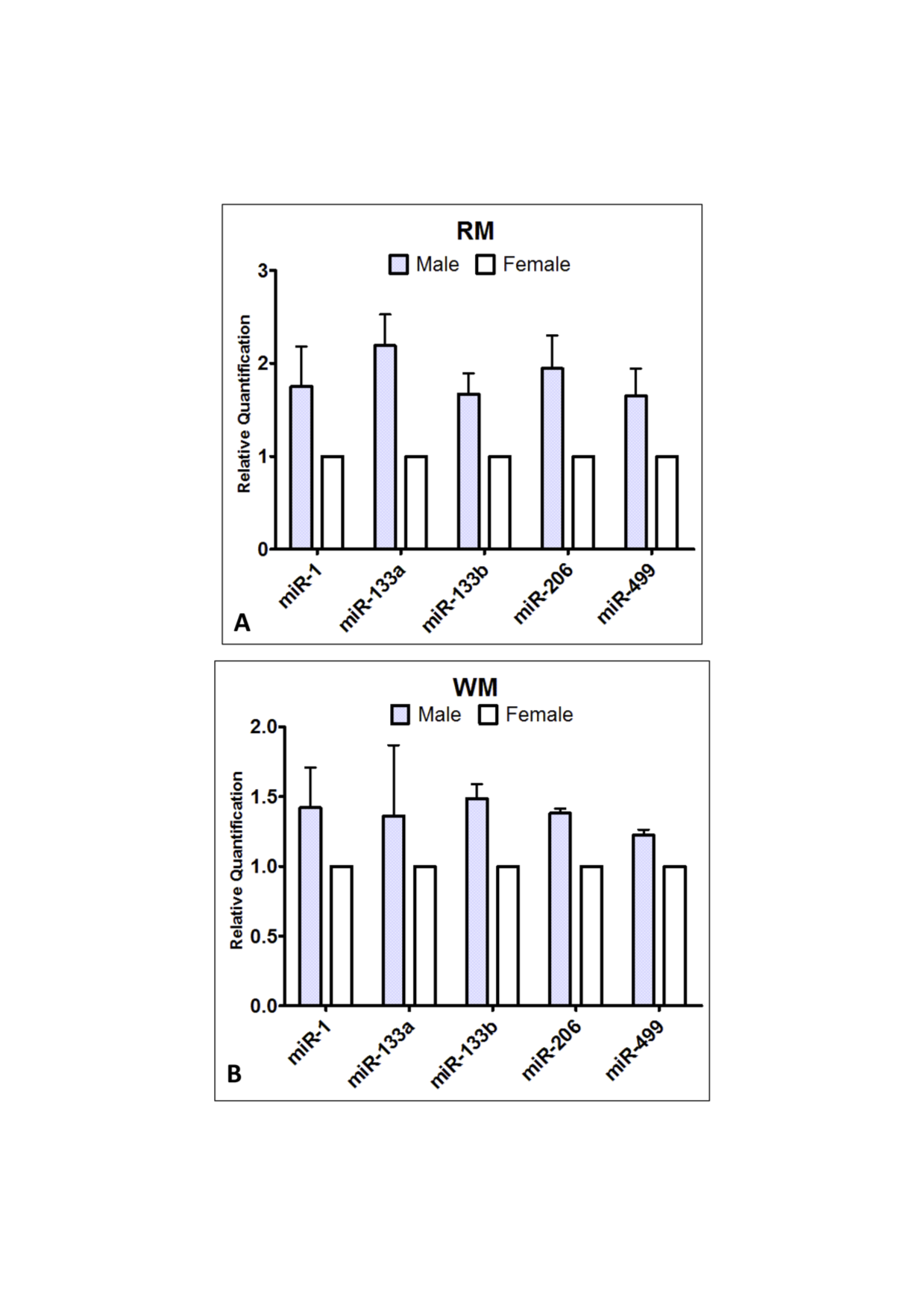

Supplement: S1 Fig — RT-qPCR shows gene expression levels of myomiRs miR-1, -133a, -133b, -206, and -499 between males and females in red muscle (A) and white muscle (B). RM = red muscle; WM = white muscle. (TIF) [file pone.0119804.s001.tif]

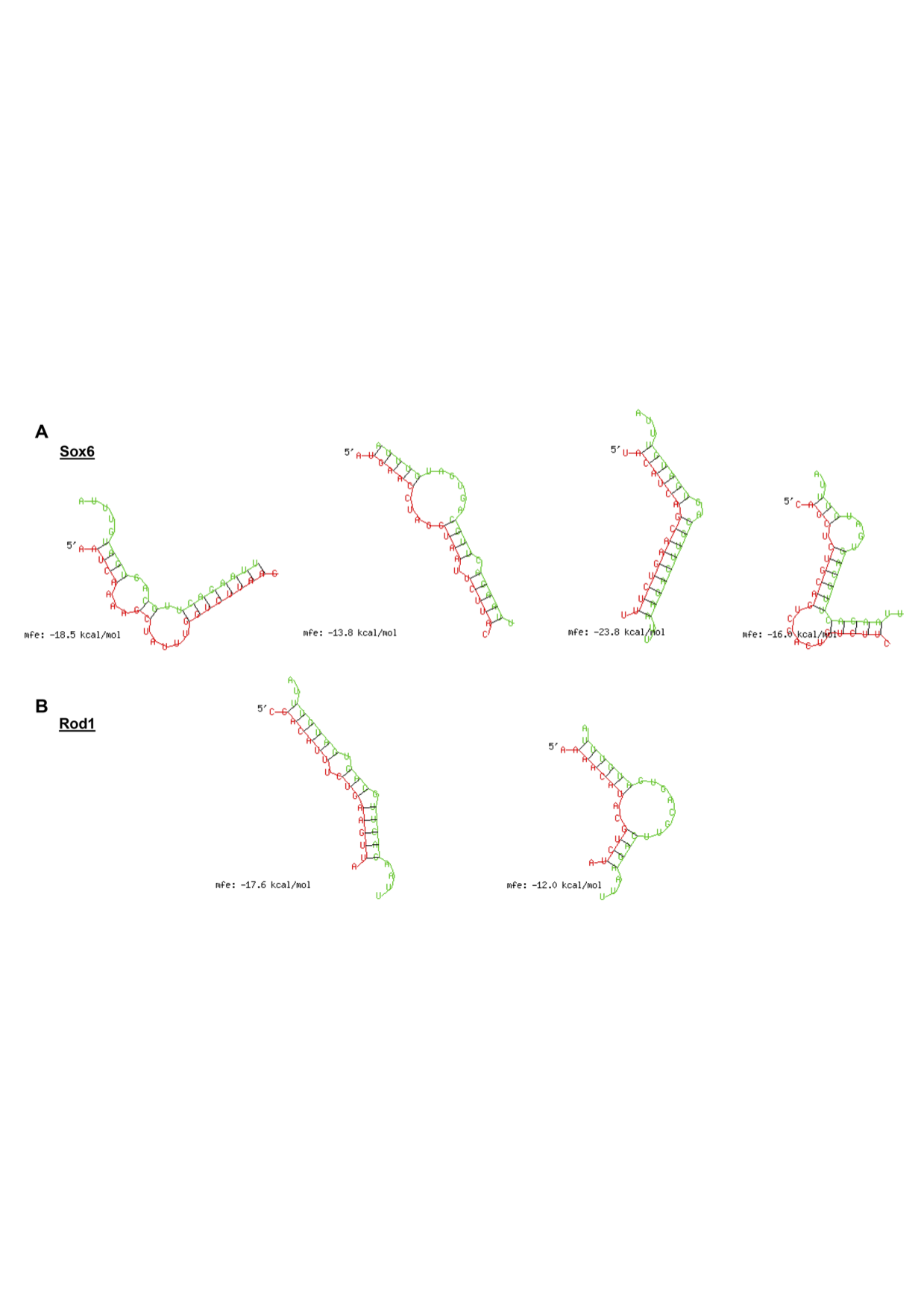

Supplement: S2 Fig — Minimum free energy (MFE) in which green lines represents the miR-499 sequence and red lines represent 4 microRNA recognition elements (MRE) on sox6 mRNA (A) or 2 MREs on rod1 mRNA (B). (TIF) [file pone.0119804.s002.tif]

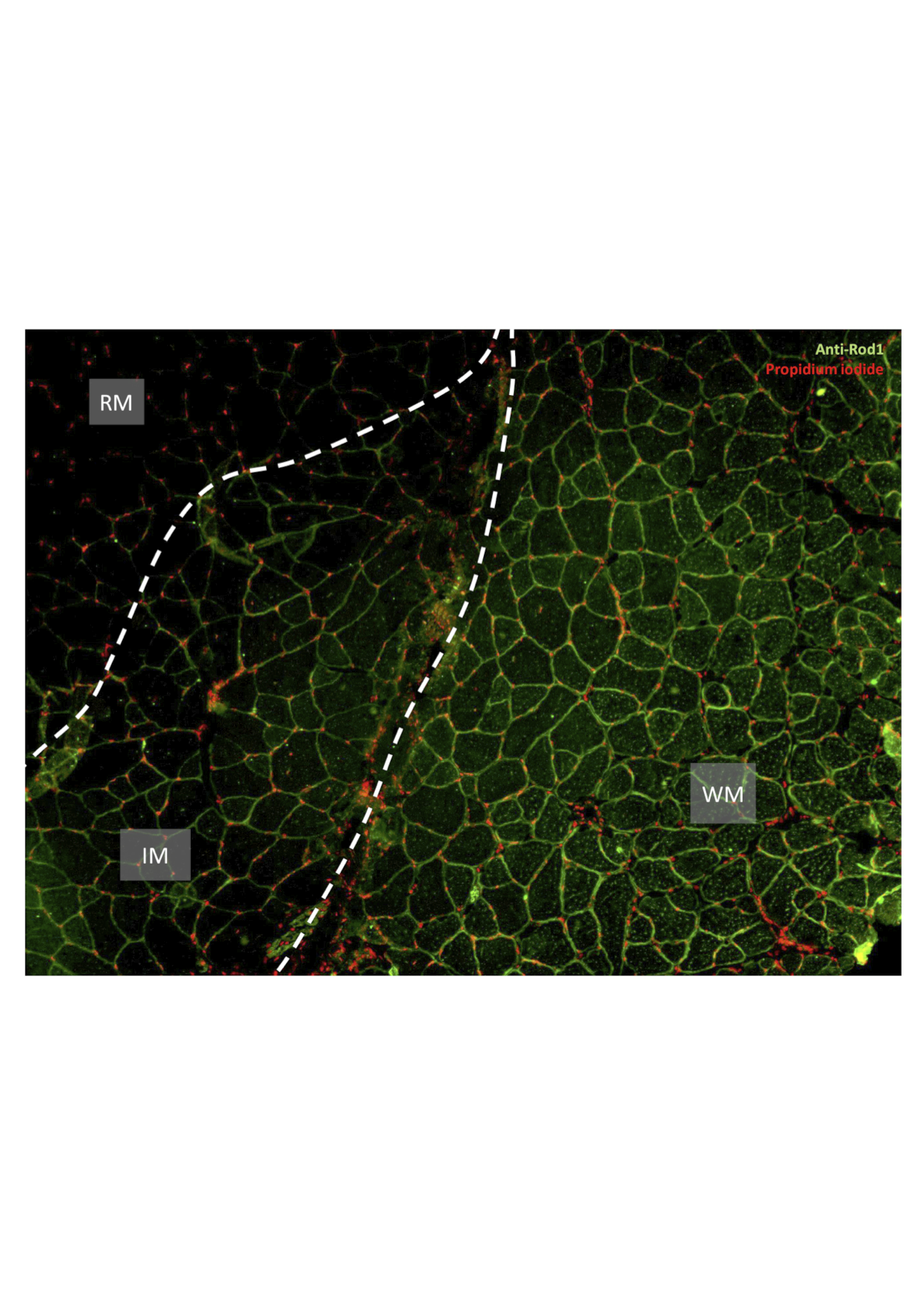

Supplement: S3 Fig — Fluorescent immunostainning demonstrates the difference of ROD1 expression in red, intermediate, and white muscles, 200x magnification. RM = red muscle; IM = intermediate muscle; WM = white muscle. (TIF) [file pone.0119804.s003.tif]

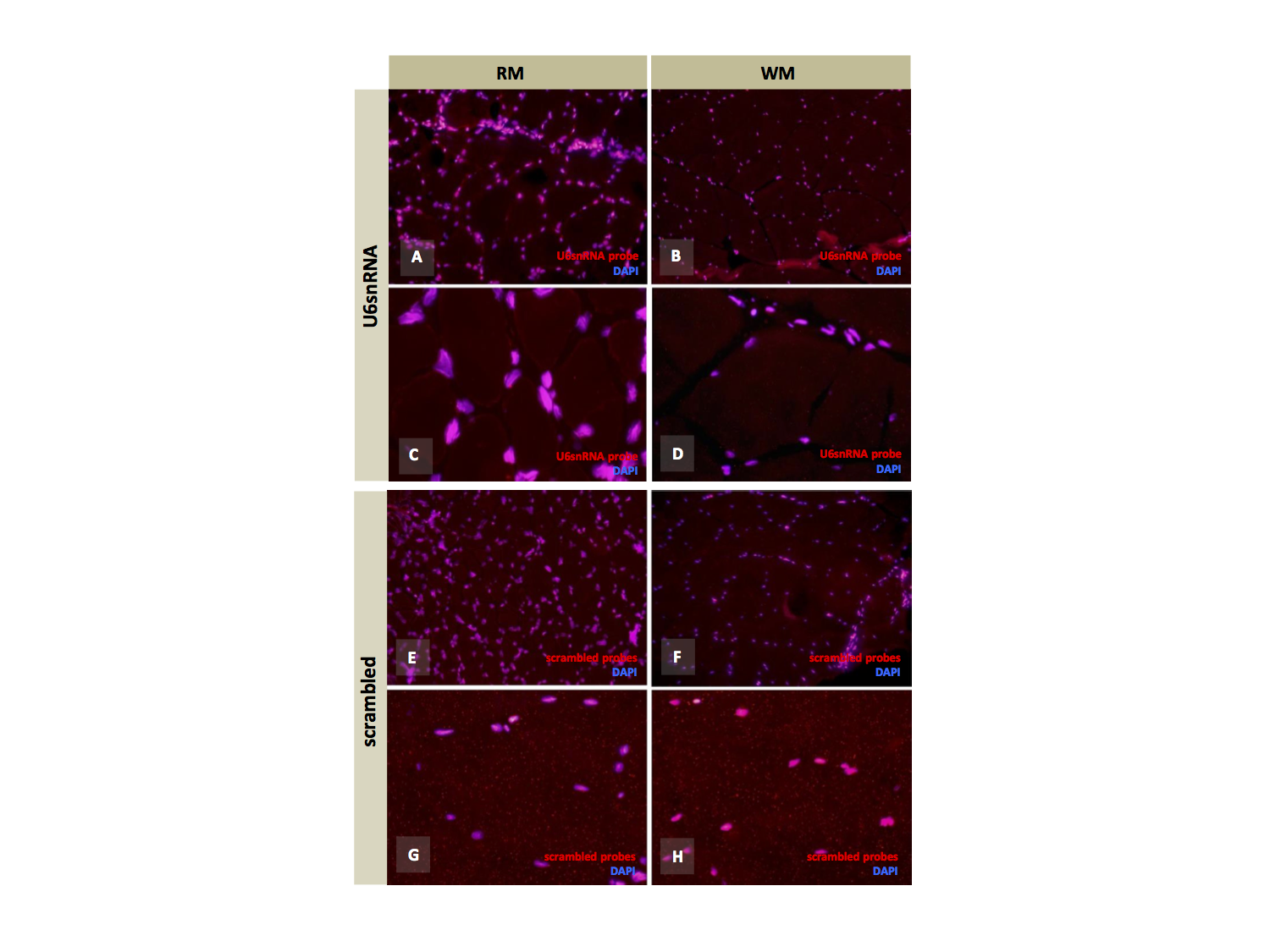

Supplement: S4 Fig — U6 snRNA and scramble LNA probes detection at red (RM) and white (WM) muscles. (TIF) [file pone.0119804.s004.tif]

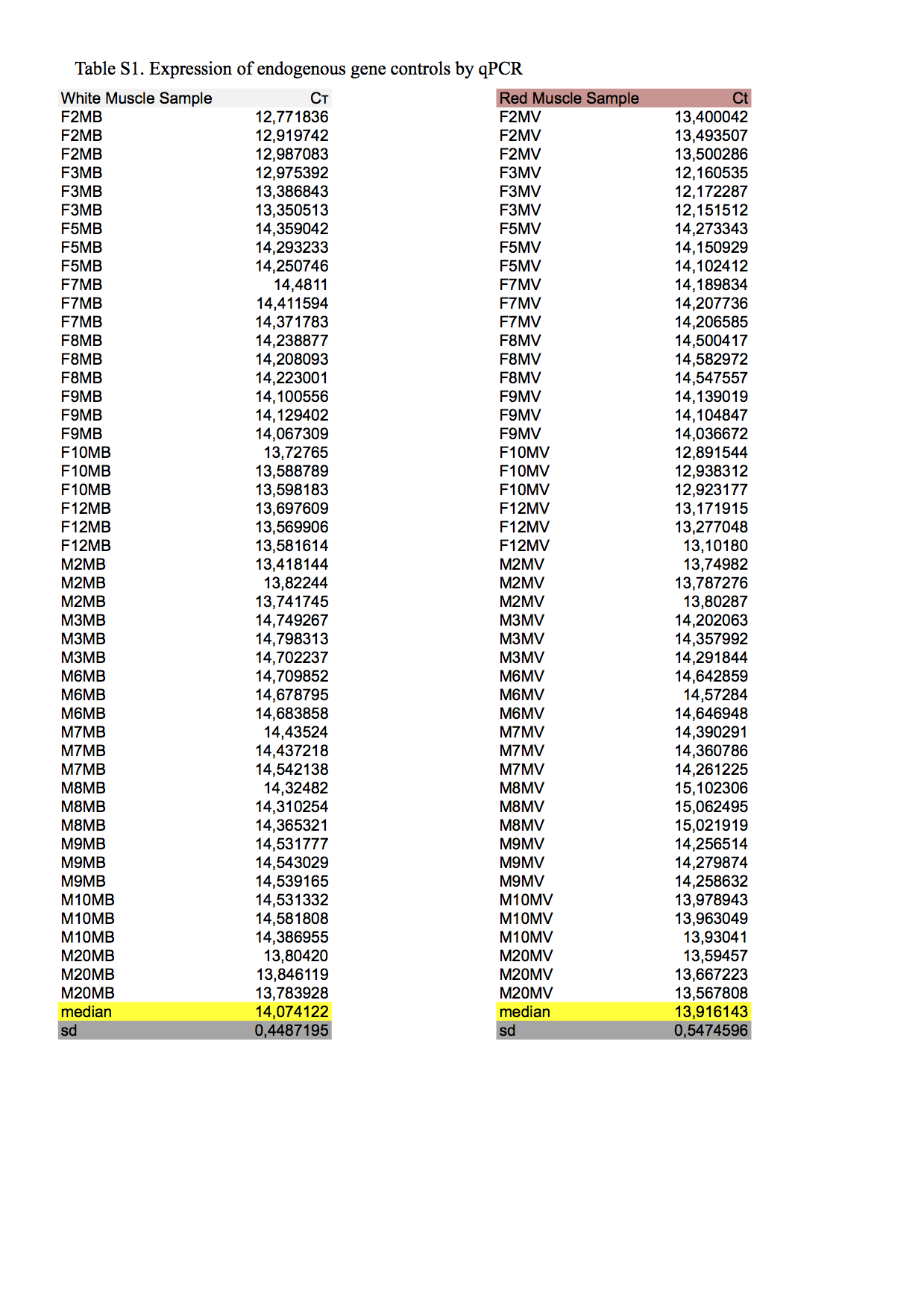

Supplement: S1 Table — U6 snRNA and 18S rRNA were equivalently expressed in red and white skeletal muscle of Nile tilapia. (TIF) [file pone.0119804.s005.tif]

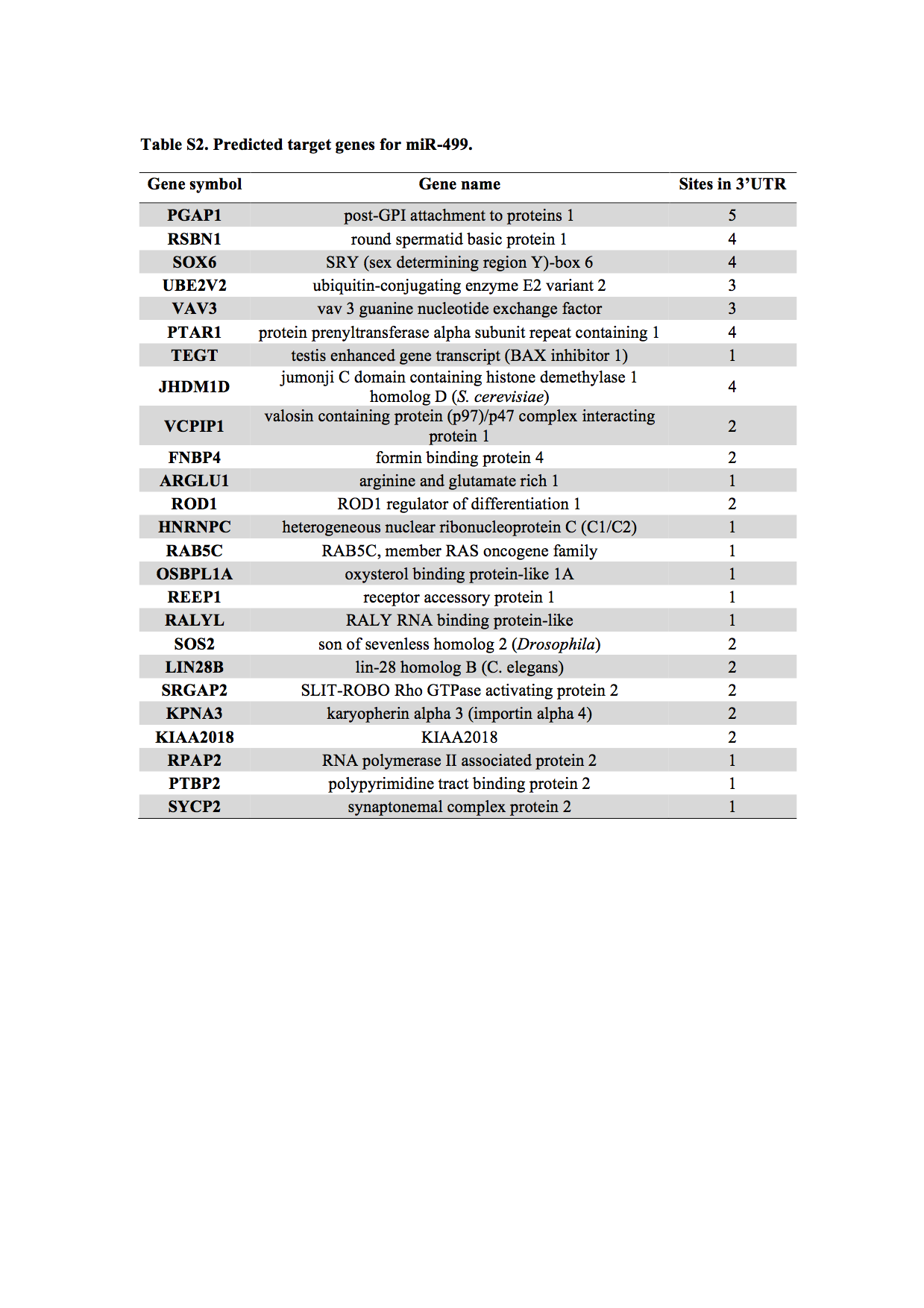

Supplement: S2 Table — List of 20 muscle-related predicted target genes for miR-499 in the human genome. (TIF) [file pone.0119804.s006.tif]

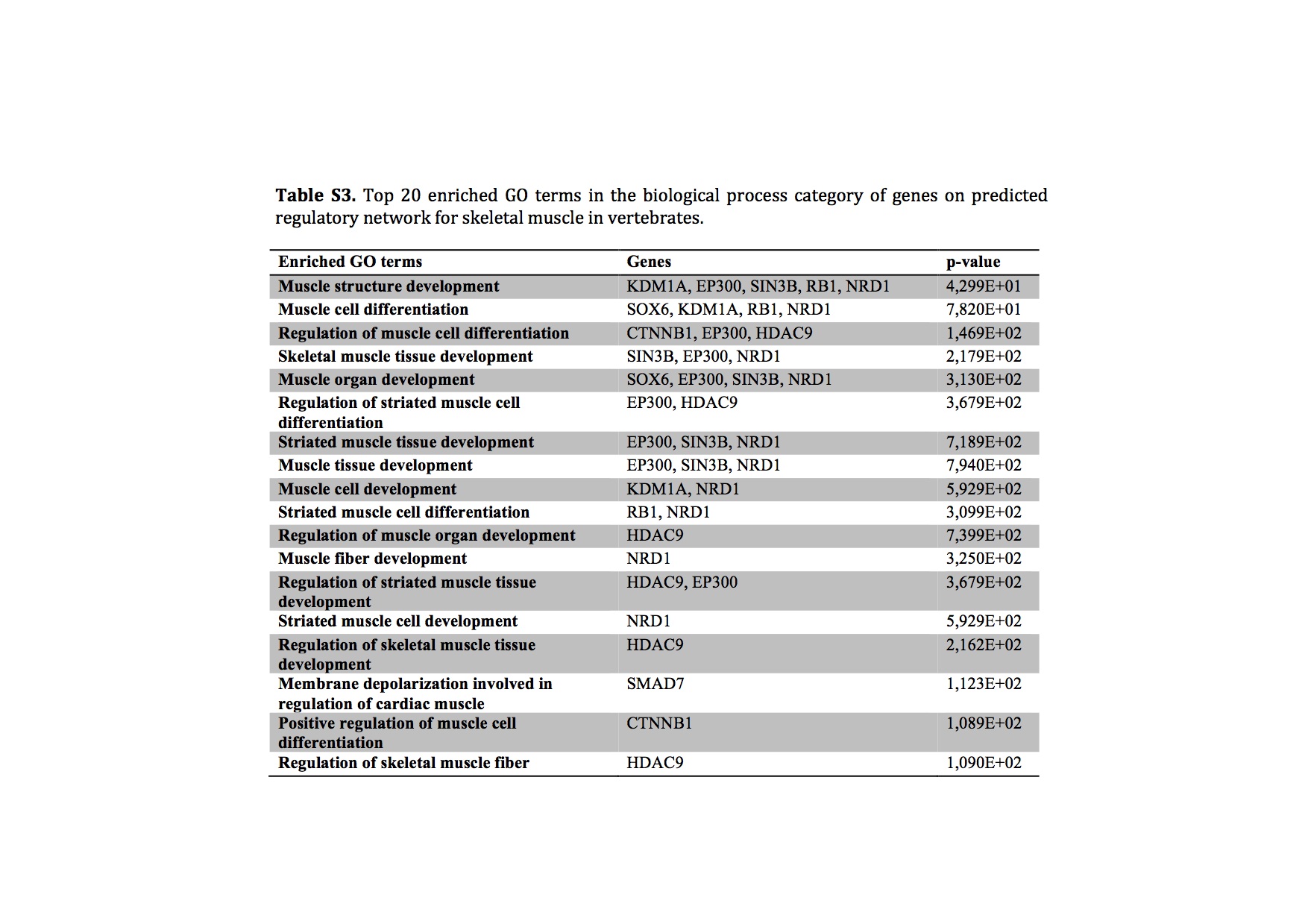

Supplement: S3 Table — Gene Ontology terms and corresponding genes related to predicted miR-499 gene regulatory network in vertebrates. (TIF) [file pone.0119804.s007.tif]

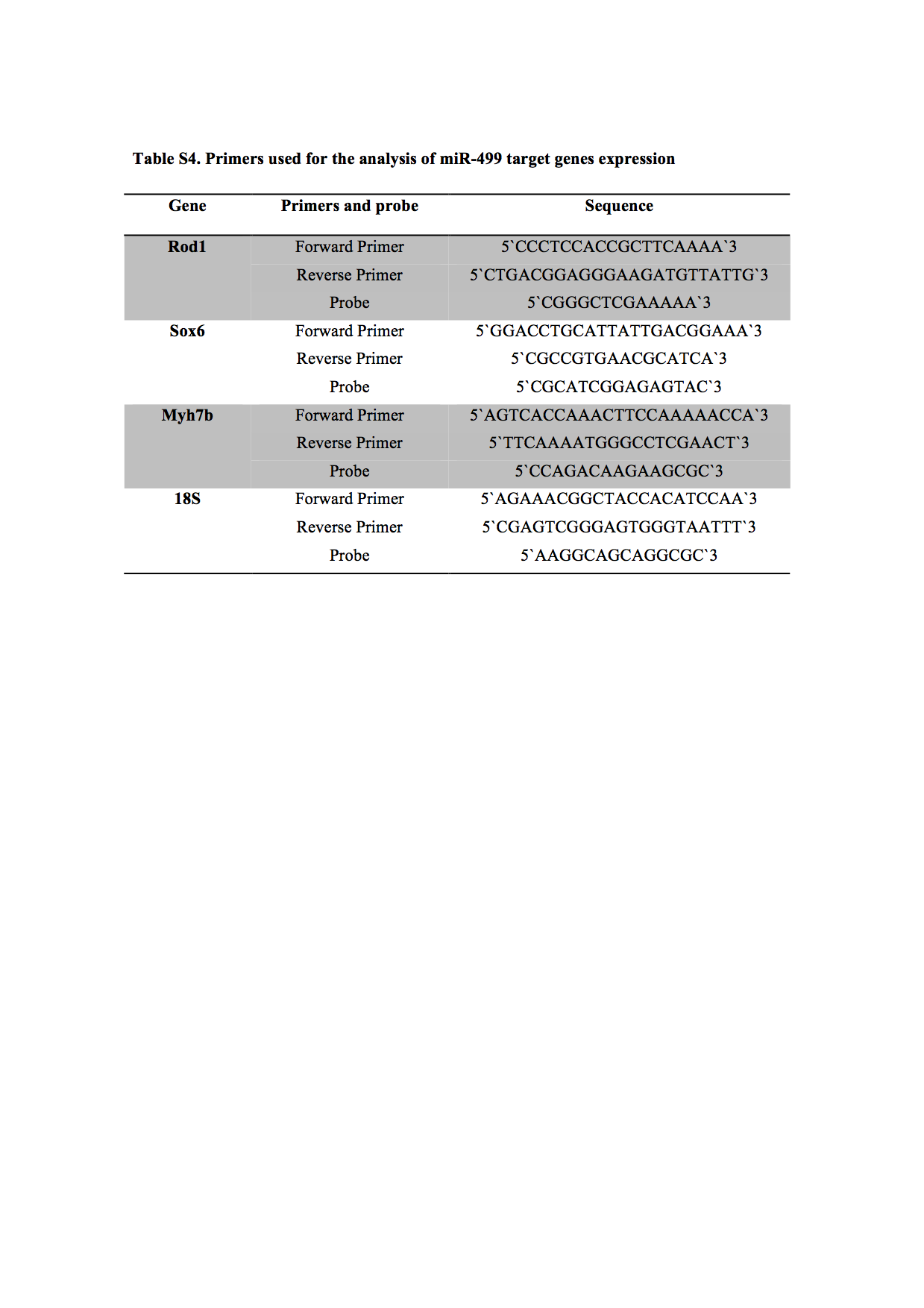

Supplement: S4 Table — (TIF) [file pone.0119804.s008.tif]

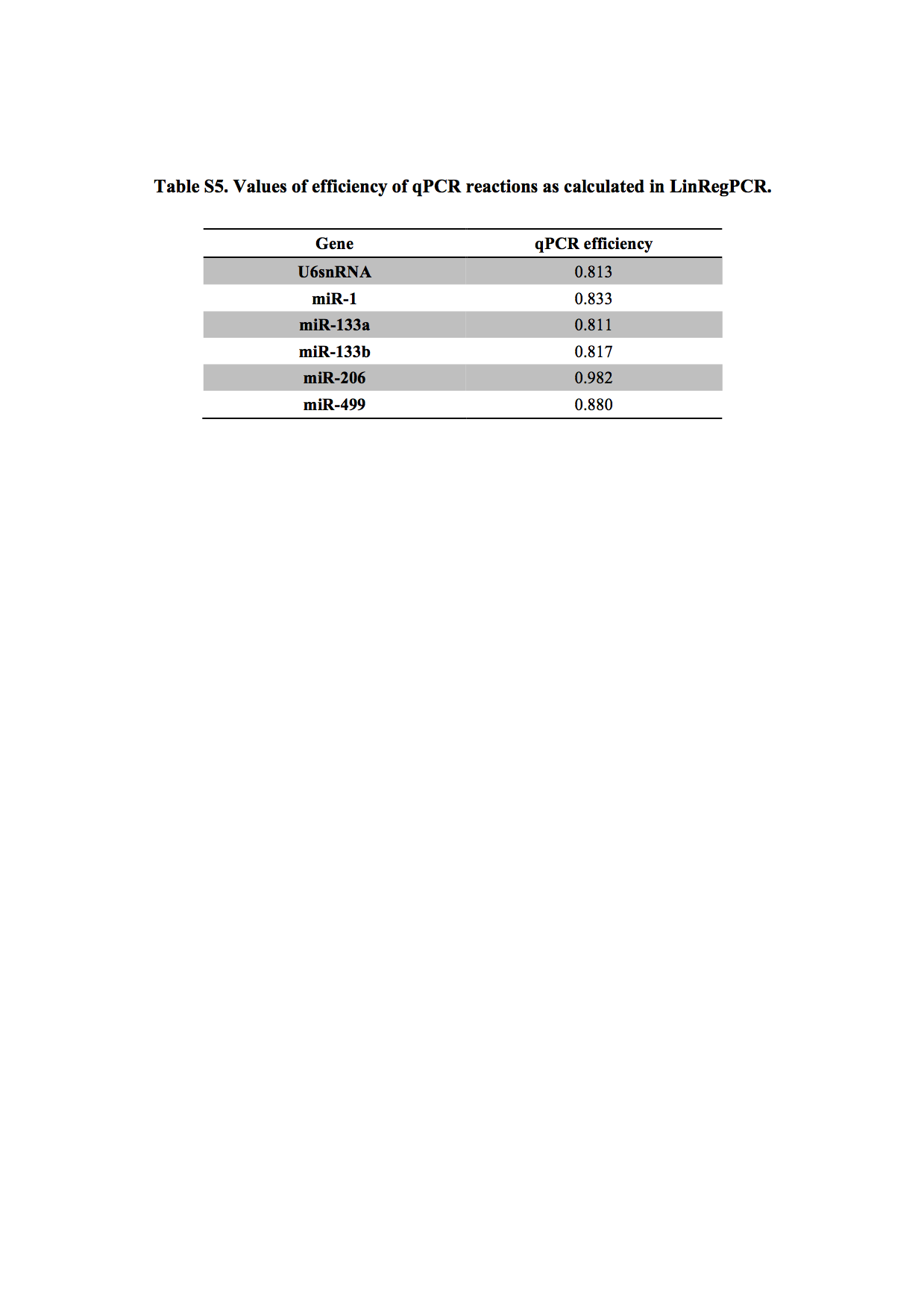

Supplement: S5 Table — (TIF) [file pone.0119804.s009.tif]
